# Editorial: The Role of the Fetal Membranes in Pregnancy and Birth

**DOI:** 10.3389/fphys.2021.653084

**Published:** 2021-02-18

**Authors:** Ramkumar Menon, Martha Lappas, Tamas Zakar

**Affiliations:** ^1^Department of Obstetrics and Gynecology/Cell Biology, The University of Texas Medical Branch Galveston, Galveston, TX, United States; ^2^Obstetrics, Nutrition and Endocrinology Group, Department of Obstetrics and Gynaecology, University of Melbourne, Heidelberg, VIC, Australia; ^3^Mercy Perinatal Research Centre, Mercy Hospital for Women, Heidelberg, VIC, Australia; ^4^Faculty of Health and Medicine, School of Medicine and Public Health, The University of Newcastle, Callaghan, NSW, Australia; ^5^Department of Maternity and Gynaecology, Hunter Medical Research Institute, John Hunter Hospital, New Lambton Heights, NSW, Australia

**Keywords:** fetal membranes, PPROM, preterm birth, amniochorion, pregnancy

Fetal membranes or amniochorionic membranes are one of the most intriguing tissues in the intrauterine cavity that are essential for the protection of the fetus, maintenance of pregnancy, and as a signaler to initiate parturition (Menon et al., 2018). However, the structure, biology, life cycle, and functions of the fetal membranes are unclear to many in the field of reproductive biology. Hence, several knowledge gaps exist that have hindered the advancement of pregnancy research as well as designing strategies to mitigate the dysfunction of this tissue, frequently associated with adverse pregnancy outcomes. This is partly because fetal membranes are often considered a mere appendage of the placenta, or even dead tissue at fetal delivery. Consequently, fetal membrane research has often been misguided, ignored, and vastly unfunded in reproductive biology and medicine (Menon and Moore, 2020). Ironically, successes in branches of medicine outside of reproductive biology, like regenerative medicine utilizing the stem cell-like properties, survivability past placental delivery, and transitional features of amniochorion cells have generated vigorous renewed interest to further understand this tissue (Martin et al., 2019).

Developmentally, fetal membranes are composed of two separate layers of tissues (Figure 1); (1) a single cell epithelial layer of amnion that forms the innermost lining of the uterine cavity, bathed in amniotic fluid and in constant touch with the fetus, and (2) the outer chorion trophoblast layer that forms the feto-maternal interface barrier by lining the maternal decidua (Menon et al., 2018). This interface is distinct from the placental-decidual interface and often not studied. The amniochorion layers are connected through a collagen rich extracellular matrix that contains amnion and chorion mesenchymal cells. Amnion and chorion layers start their growth and development at the time of implantation from distinct lineages. The amnion is derived from extraembryonic somatic mesoderm and epiblast-derived amnioblasts whereas the chorion is formed by extraembryonic mesoderm and trophoblasts (Luckett, 1978; Rasweiler, 1990; Carter, 2016). The amnion and the chorion fuse to become a single unit structure around the late 1st or early 2nd trimester to shape up the intrauterine cavity (Ulm et al., 1999). Amniochorion provides mechanical, immune, endocrine, transport, and antimicrobial functions during pregnancy. Fetal membranes age as gestation progresses and it is correlated with fetal growth and development (Menon and Richardson, 2017; Menon et al., 2020). As longevity of the membranes approaches its limit at term, membranes show aging pathology (dysfunctions) highlighted by inflammation. Inflammatory mediators from aging fetal membranes are among the key fetal biological signals to initiate parturition. Thus, fetal membranes help to maintain

**Figure 1:**
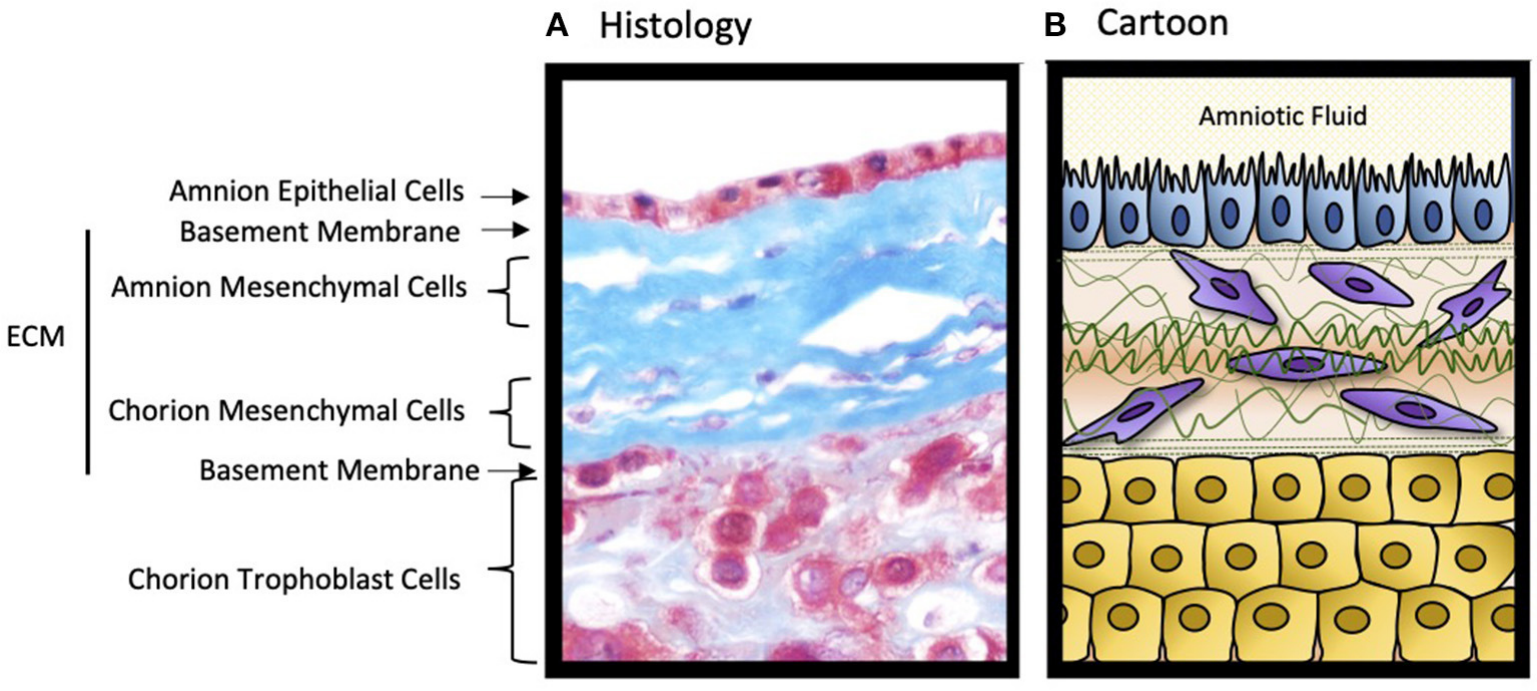
The structure of the human fetal membranes. **(A)** Microscopic cross section of the fetal membrane tissues stained with Masson's trichrome. **(B)** Cartoon representation of the histological image. The decidua (not shown) is fused to the chorion trophoblast cell layer forming the feto-maternal interface. ECM, extracellular matrix. The illustration has been created by Dr. Lauren Richardson.

pregnancy as well as promote parturition (Menon et al., 2020). Compromising the cellular and matrix architecture of the membranes can be detrimental contributing to various adverse pregnancy outcomes, particularly preterm premature rupture of the membranes (pPROM) and spontaneous preterm birth (PTB). Rupture of the membranes at term is a physiologic requirement for feto-placental delivery; however, pPROM is associated with ~40% of all PTB. Currently, there are no diagnostic indicators for these conditions and early delivery is the best available strategy to avoid complications to the developing fetus. A better understanding of fetal membrane functions *in utero*, mechanisms underlying its longevity, and cellular biological properties are essential to determine the pathological complications contributing to its dysfunctions and adverse pregnancy outcomes.

The challenges to understand fetal membrane functions and dysfunctions are substantial. A major issue is the impracticality of obtaining fetal membrane tissues during pregnancy unless delivered preterm. In addition, pregnancy complications associated with preterm deliveries can confound membrane studies. Animal models often do not mimic human fetal membranes. These circumstances impede research and limit the translational impact of work in the field. To help addressing the needs of interested scientists, the Fetal Membrane Society (FMS https://www.fetalmembranesociety.org/) was formed in 2019. This Research Topic has been assembled in response to encouragement by the FMS to highlight the latest data and chart future prospects in fetal membrane research.

The collection includes 16 articles covering the basic, functional, and translational aspects of fetal membrane biology to show how these tissues contribute to pregnancy and pregnancy associated pathologies. Three manuscripts discuss the *genomics, epigenomics, and proteomics* of fetal membranes. Cunningham et al. and Zakar and Paul reviewed articles on fetal membrane genome, exome, methylome, and epigenome and displayed differences in the expression of various functional molecules in normal and abnormal pregnancies. Proteomics analysis of fetal membranes from normal and preterm pregnancies was discussed by Pan et al. showing how inflammatory pathways and collagen metabolisms are impacted in preterm births either with or without pPROM. Fetal membranes are a rich source of various *endocrine mediators and their receptors*. The roles of fetal membrane-derived eicosanoids and exosomes in human parturition are discussed by Mosaad et al. Further, endocrine mediators support cellular and matrix homeostasis. Kang Sun's group has contributed immensely to the knowledge of cortisol regeneration in fetal membrane matrix (mesenchymal) cells by 11β-hydroxysteroid dehydrogenase 1 (11β-HSD1). A review on this topic is provided by Wang et al. which discusses the physiologic and pathologic contributions of 11β-HSD1 in fetal membranes and its contributions to preterm and term parturitions. Another key mediator that maintains fetal membrane homeostasis during pregnancy is progesterone. Marinello et al. describes how amnion membrane matrix mesenchymal cells use cell membrane-associated progesterone receptors in providing a progesterone dependent anti-inflammatory environment during pregnancy.

Like all other tissues, fetal membranes start aging *in utero* and aging progresses during gestation. Premature *senescence of the fetal membranes* leading to dysfunction is one of the reasons underlying pPROM and PTB. The review by Polettini and Guimarães da Silva focuses on the telomere-dependent aging of fetal membranes and its association with telomerase dynamics.

Fetal membrane pathologies are often associated with *inflammation*. One of the classic signs that describe the severity of adverse pregnancy outcomes and a factor often used to design management strategies of preterm neonates is based on the histologic assessment of fetal membrane inflammation. Infiltration by neutrophils, termed histologic chorioamnionitis, is an indicator of the severity of inflammation in the intrauterine cavity. Compromising immune defense is one of the mechanisms causing fetal membrane inflammation, immune cell infiltration, and membrane dysfunctions leading to chorioamnionitis-associated preterm birth or pPROM. *Exogenous risk exposures* and their interactions with endogenous factors like the microbiome often amplify pregnancy risk. An article about maternal environmental pollutant exposures that can weaken innate immune defense is discussed by Harris et al. In this review, authors report risk modification by an interaction between the environment and pathogenic vaginal colonizers such as Group B streptococcus. Wheeler and Oyen review data related to the interesting hypothesis that fetal membranes may actually behave as an inflated balloon, and pressure changes during severe weather events can promote pPROM. Inflammation and inflammatory cytokines are well-studied in the fetal membranes. A report by Pardon et al. in this Research Topic and multiple reports by Vincent Sapin and Loïc Blancho's group have provided novel ideas to this area by discussing the roles and contributions of *damage associated molecular patterns* (DAMPs) and their receptors. By analyzing DAMPs and their receptors on *maternal* biological samples and exosomes, Sapin's group report a lack of association between these markers and pPROM. As reported, high abundance of DAMPs in *fetal* membranes and compartments is suggestive of fetal contributions to preterm birth and pPROM.

An understudied area of fetal membrane research is its *growth, remodeling, and repair*. A better understanding of these mechanisms can improve outcomes of fetoscopy and fetal surgeries as these procedures often contribute to membrane rupture and preterm delivery. Based on their own reported findings, Mogami and Word summarize how epithelial mesenchymal transition (EMT) of amnion epithelial cells can be a mechanism in fetal membranes that can heal membrane wounds. Approaches to enhance this process are also discussed. Multiple factors have been described to promote EMT in various cells. One of them is IL-6, a cytokine produced in high abundance during fetal membrane dysfunctions. IL-6 has been considered one of the cytokines contributing to preterm birth and pPROM as its levels are very high in all pregnancy associated biological fluids and tissues. A basic science report is included in this Research Topic by Omere et al. where they studied IL-6's effect on amnion epithelial cells. IL-6 lacks cell fate determining properties even at levels seen during pPROM or PTB and it does not appear to promote EMT in fetal membrane cells.

Lack of biomarkers indicative of membrane dysfunction hampers clinical management of membrane-associated complications such as pPROM and spontaneous preterm birth. An innovative approach using MRI has been introduced recently by Wang et al. to determine membrane pathologies. The potential of this approach as an *imaging-based biomarker* to test membrane function is reviewed by Qi et al. In their report, Omere et al. also describe the role of IL-6 as a potential biomarker associated with adverse events, although IL-6 does not seem to have any specific impact on fetal membrane cells.

Fetal membrane research is notoriously obstructed by a lack of specialized techniques and tools. Richardson et al. points out how innovative approaches like fetal membrane *organ on a chip* (OOC) can be used to replace traditional 2D cell cultures, transwells, and organ explant models to study fetal membrane biology and function. In their review, three phases of fetal membrane OOCs development have been outlined and its advantages and disadvantages are discussed. Without a doubt, the *in vitro* reconstruction of fetal membrane tissues is an exciting futuristic approach with great potential.

In summary, the Research Topic “The Role of the Fetal Membranes in Pregnancy and Birth” provides a comprehensive overview of fetal membrane biology, endocrine, mechanical, immune functions, normal and risk-associated changes to the membranes, development of biomarkers to diagnose membrane functions or dysfunctions, and tools to study fetal membranes. The collection of papers, under the auspices of the Fetal Membrane Society, will certainly promote fetal membrane biology to take its deserved place of prominence in perinatal science.

## Author Contributions

RM, ML, and TZ contributed to this editorial. All authors contributed to the article and approved the submitted version.

## Conflict of Interest

The authors declare that the research was conducted in the absence of any commercial or financial relationships that could be construed as a potential conflict of interest.
